# Importance of long non-coding RNAs in the pathogenesis, diagnosis, and treatment of prostate cancer

**DOI:** 10.3389/fonc.2023.1123101

**Published:** 2023-03-21

**Authors:** Mohammad Taheri, Elham Badrlou, Bashdar Mahmud Hussen, Amir Hossein Kashi, Soudeh Ghafouri-Fard, Aria Baniahmad

**Affiliations:** ^1^ Institute of Human Genetics, Jena University Hospital, Jena, Germany; ^2^ Urology and Nephrology Research Center, Shahid Beheshti University of Medical Sciences, Tehran, Iran; ^3^ Men’s Health and Reproductive Health Research Center, Shahid Beheshti University of Medical Sciences, Tehran, Iran; ^4^ Department of Clinical Analysis, College of Pharmacy, Hawler Medical University, Erbil, Kurdistan, Iraq; ^5^ Department of Medical Genetics, School of Medicine, Shahid Beheshti University of Medical Sciences, Tehran, Iran

**Keywords:** lncRNA, prostate cancer, biomarker, expression, diagnostic

## Abstract

Long non-coding RNAs (lncRNAs) are regulatory transcripts with essential roles in the pathogenesis of almost all types of cancers, including prostate cancer. They can act as either oncogenic lncRNAs or tumor suppressor ones in prostate cancer. Small nucleolar RNA host genes are among the mostly assessed oncogenic lncRNAs in this cancer. PCA3 is an example of oncogenic lncRNAs that has been approved as a diagnostic marker in prostate cancer. A number of well-known oncogenic lncRNAs in other cancers such as DANCR, MALAT1, CCAT1, PVT1, TUG1 and NEAT1 have also been shown to act as oncogenes in prostate cancer. On the other hand, LINC00893, LINC01679, MIR22HG, RP1-59D14.5, MAGI2-AS3, NXTAR, FGF14-AS2 and ADAMTS9-AS1 are among lncRNAs that act as tumor suppressors in prostate cancer. LncRNAs can contribute to the pathogenesis of prostate cancer *via* modulation of androgen receptor (AR) signaling, ubiquitin–proteasome degradation process of AR or other important signaling pathways. The current review summarizes the role of lncRNAs in the evolution of prostate cancer with an especial focus on their importance in design of novel biomarker panels and therapeutic targets.

## Introduction

Prostate cancer is the most commonly diagnosed cancer among males being responsible for 27% of all diagnosed cases (1). It also accounts for the greatest number of deaths from cancer among men after lung cancer (1). A number of risk factors have been identified for prostate cancer among them are age, ethnicity, genetics, family history, obesity, and smoking (2, 3). Prostate cancer is developed *via* a multistep process, starting from prostatic intraepithelial neoplasia and being evolved to localized, advanced prostate cancer with local invasion and metastatic prostate cancer, respectively (4). The aggressiveness of prostate cancer is best described by the Gleason grading system (5). The hormone responsiveness is an important feature in this cancer resulting in tumor regression following castration (6). Therefore, androgen deprivation therapy has been suggested as the regular therapeutic regimen for prostate cancer. However, resistance to this therapeutic modality can develop (4).

Identification of the underlying cause of initiation and progression of prostate cancer is an imperative step in development of novel therapies for this kind of malignancy. Moreover, it can facilitate design of novel biomarkers for early detection of cancers. Long non-coding RNAs (lncRNAs) are promising transcripts for both purposes (7–9). These transcripts have sizes more than 200 nucleotides and are responsible for a variety of regulatory mechanisms at different levels of gene expression regulation (10). Aberrations in the expression of lncRNAs might be representative of certain phases of cancer progression, and can be used to predict early progression of cancer or induction of cancer‐related signaling pathways (11, 12). Therefore, these transcripts have attained much attention during recent years for their contribution in the pathogenesis of almost all kinds of cancers, including prostate cancer. The current review summarized the role of lncRNAs in the evolution of prostate cancer with an especial focus on their importance in design of novel biomarker panels and therapeutic targets. We used PubMed and Google Scholar databases with the key words “lncRNA” or “long non-coding RNA” and “prostate cancer”. Then, we screened the obtained articles and included the relevant ones in the manuscript. Finally, we tabulated the data obtained from these articles for the purpose of better classification of the data.

## Up-regulated lncRNAs in prostate cancer

Using quantitative real time PCR method, several lncRNAs have been shown to be over-expressed in prostate cancer tissues compared with adjacent non-cancerous tissues or benign prostate hyperplasia (BPH) samples, representing an oncogenic role for these transcripts in the progression of prostate cancer (**Table 1**). Small nucleolar RNA host genes (SNHGs) are among the mostly assessed lncRNAs in this field. A number of well-known oncogenic lncRNAs in other cancers such as DANCR, MALAT1, CCAT1, PVT1, TUG1 and NEAT1 have also been shown to act as oncogenes in prostate cancer. For instance, DANCR has been found to contribute to the taxol resistance of in this type of cancer *via* modulation of miR-33b-5p/LDHA axis (44). Expression of this lncRNA has been up-regulated in serum samples of prostate cancer patients, parallel with down-regulation of miR-214-5p. Notably, DANCR expression has been correlated with PSA level, Gleason score and T stage in these patients. DANCR expression not only can be used for prostate cancer diagnosis, but also can predict poor prognosis of this type of cancer with high diagnostic value. Mechanistically, up-regulation of DANCR or down-regulation of miR-214-5p could enhance proliferation and migration, preclude apoptosis, and induce activity of TGF-β signaling (45). DANCR can also target miR-185-5p to increase expression of LIM and SH3 protein 1 promoting prostate cancer through the FAK/PI3K/AKT/GSK3β/snail axis (46).

**Table 1:** Summary of function of up-regulated lncRNAs in prostate cancer (Official HUGO Gene Nomenclature symbols are used).

lncRNA	Samples	Cell lines	Targets/Regulators	Signaling Pathways	Association with patients’ outcome	Function	Ref
**UBE2R2-AS1**	74 PTNTs	RWPE-1, DU145, and PC-3	PCNA, CDK4, Cyclin D1, Bcl-2, N-cadherin, Vimentin, E-cadherin	–	Poor prognosis of PC patients	Might serve as a biomarker for diagnosis and a promising target in case of PC therapy	(13)
**CASC11**	66 PTNTs	PC-3, DU145, 22Rv1, LNCaP, and RWPE-1	YBX1	p53 pathway	–	CASC11 enhances the proliferation and migratory capacity of PC cells.	(14)
**CASC11**	29 tumor and 5 benign prostate samples	PNT1a, PC3, DU145, and LNCaP	miR-145	PI3K/AKT/mTOR and CASC11/miR-145/IGF1R axis	–	Its high expression suppresses miR-145, and activates PI3K/AKT/mTOR pathway.	(15)
**SNHG17**	52 PTNTs	RWPE-1, RV-1, PC-3, DU145, and LNCaP	miR-23a	SNHG17/miR-23a/OTUB1 Axis	Advanced tumor stage	SNHG17 may enhance the progression of PC.	(14)
**SNHG17**	58 PTNTs	LNCaP, C4-2, and HPrEC	TCF1, TCF4, LEF1, c-myc, cyclin D1 and axin2	Wnt/β-catenin pathway	Poor outcomes	SNHG17 promotes the proliferation and viability, but suppresses apoptosis.	(16)
**SNHG17**	36 PTNTs	RWPE-1, DU145, LNCaP, VCaP, and PC-3	SNORA71B, miR-339-5p, and STAT5A	SNHG17/miR-339-5p/STAT5A/SNORA71B axis	Low PFS	SNHG17/miR-339-5p/STAT5A modulates SNORA71B expression.	(17)
**SNHG17**	46 patients with CRPC and 149 patients with HSPC	LNCaP, C4-2, PC-3, and DU145	miR-144 and CD51	miR-144/CD51 Axis	–	Expression of SNHG17 was elevated in CRPC tissues and cells.	(18)
**SNHG16**	80 PTNTs	DU-145 PCa cells	miR-373-3p	TGF-β-R2/SMAD signaling	–	SNHG16 facilitates the proliferation and migration by modulating the miR-373-3p/TGF-β-R2/SMAD axis.	(19)
**SNHG16**	52 cancer tissues and 36 normal prostate samples	22Rv1 and HPrEC	GLUT1	–	–	SNHG16 silencing suppresses the growth of PCa cells through downregulating GLUT1.	(20)
**SNHG14**	60 PTNTs	WPMY1, LNCaP, 22RV1, PC-3, and DU145	miR-5590-3p, YY1, Cyclin D1, Bcl-2, N-cadherin, Bax, Caspase-3, and E-cadherin	miR-5590-3p/YY1 axis	Advanced stage and poor diagnosis	SNHG14 enhances the proliferation and invasion of PCa cells through miR-5590-3p/YY1.	(21)
**SNHG12**	85 PTNTs	WPMY-1, LNCAP, DU145, and PC-3	apoptosis-related and invasion-related proteins	PI3K/AKT signaling pathway	–	SNHG12 Silencing suppresses PCa cells proliferation.	(22)
**SNHG12**	Blood samples from 56 PCa patients and 45 patients with BPH	22RV1, Du145, LNCaP, MDaPCa2b, and RWPE1	CCNE1 and miR-195	PI3K/AKT/mTOR pathway and miR-195/CCNE1 axis	Poor prognosis	SNHG12 silencing suppresses viability and induces apoptosis and autophagy of PCa cells.	(23)
**SNHG11**	120 PCa patients and 45 cases of BPH patients	22RV1	–	–	Shorter OS time and biochemical recurrence-free survival	SNHG11 silencing prevents the proliferation, invasion, and migration.	(24)
**SNHG11**	30 PTNTs	RWPE-1, LNCaP, C4-2, PC3, and DU145	miR-184	miR-184/IGF-1R signaling axis	–	SNHG11 promotes progression of PC by increasing the expression of IGF-1R.	(25)
**SNHG10**	gene expression profiles of PC patients from TCGA database	VCaP, LNCaP, 22RV1, PC3, DU145, and RWPE-1	–	Immune infiltration and oxidative phosphorylation	Advanced clinical parameters	SNHG10 affects proliferation, migration, and invasion.	(26)
**SNHG9**	52 PTNTs	–	–	maintenance of cell metabolism and protein synthesis	Poor prognosis	SNHG9 may serves as a possible prognostic biomarker in patients with PCa.	(27)
**SNHG8**	53 PTNTs	RWPE1, LNCaP, PC3, DU145, VCap, and 22RV1	miR-384 and HOXB7	–	–	SNHG8 enhances the proliferation, migration and invasion of PCa cells by sponging miR-384.	(28)
**SNHG7**	30 PTNTs	PC-3 and DU-145 cells	c-Myc	SRSF1/c-Myc axis	–	SNHG7 knocking down inhibits the proliferation and glycolysis in PCa cells.	(29)
**SNHG7**	127 PTNTs	–	–	–	Metastasis, pelvic lymph node metastasis, and TNM stage	SNHG7 may serve as a possible prognostic marker and target for the treatment of PCa.	(30)
**SNHG6**	63 PTNTs	PC-3 and DU145	miR-186	SNHG6/miR-186 axis	–	SNHG6 was upregulated in drug-resistant PCa tissues and cells.	(31)
**SNHG3**	30 PTNTs	RWPE-1, PC-3, DU145, VCaP and LNCaP	miR-1827	Wnt/AKT/mTOR pathway	Poor prognosis	SNHG3 may be a prognostic marker for PCa.	(32)
**SNHG3**	40 PTNTs	WPMY-1, PC-3, Du 145, LNCaP, and 22RV1	miR-152-3p	SNHG3/miR-152-3p/SLC7A11 axis	–	Promotes proliferation, invasion, and migration of PCa cells *via* sponging miR-152-3p.	(33)
**SNHG3**	26 PTNTs	REPW-1, DU145, VCaP, LNCaP, C4-2B, 22RV1,and PC3	miR-214-3p	SNHG3/miR-214-3p/TGF-β axis	Advanced clinicopathological features and poor prognosis	SNHG3 silencing suppresses bone metastasis in PCa cell.	(32)
**SNHG3**	PTNTs	LNCaP and PC-3	miR-487a-3p and TRIM25	EMT	–	SNHG3 sponges with miR-487a-3p, and affects migration, invasion, and EMT of PCa cells.	(34)
**SNHG3**	–	RWPE‐1, PC3, DU145, 22RV1, and LNCaP	miR-577 and SMURF1	SNHG3/miR‐577/SMURF1 axis	–	SNHG3 affects the proliferation, migration, EMT process and apoptosis.	(35)
**SNHG1**	Formalin fixed paraffin—embedded PCa specimens and BPH or ANTs (n=14)	RWPE-1, LNCaP, 22Rv1, PC-3, DU145	E-cadherin, vimentin	EMT pathway	Tumor metastasis	SNHG1 is a possible target for treatment of PCa.	(36)
**SNHG1**	20 PTNTs	LNCaP, PC-3, DU-145, and RWPE-1	EZH2	Wnt/β-catenin and PI3K/AKT/mTOR signaling pathway	–	SNHG1 affects PCa cells proliferation, apoptosis, migration, invasion, and autophagy by targeting EZH2.	(37)
**SNHG1**	134 PTNTs	PC3 and DU145	–	–	Aggressive malignant behavior	SNHG1 may serves as a possible marker and target for treatment of PCa.	(38)
**SNHG1**	142 PTNTs	DU-145, LNCaP, 22Rv1, PC-3, and RWPE-1	miR-195-5p, E-cadherin, N-cadherin, and Vimentin	EMT	–	SNHG1 affects PCa cells proliferation, invasion and EMT *via* sponging miR-195-5p.	(39)
**SNHG1**	Normal tissues (n=318) and PCa tissues(n=92)	22Rv1 and LNCaP	miR-377-3p and AKT2	SNHG1/miR-377-3p/AKT2 axis	Poor overall survival rate	SNHG1 sponges with miR-377-3p in PCa cells.	(40)
**lncHUPC1**	70 PTNTs	RWPE-1, LNCaP, 22RV1, DU145, and PC3	FOXA1, SDCCAG3, and miR-133b	lncHUPC1/miR-133b/SDCCAG3 axis	Advanced TNM stages	lncHUPC1 acts as an oncogene and increases the metastasis and growth of PCa cells.	(41)
**MNX1-AS1**	40 PTNTs	LNCaP, PC-3, C4-2B, Du-145 and RWPE1	miR-2113	miR-2113/MDM2 axis	Worse overall survival rates	MNX1-AS1 enhances the proliferation, migration and invasion of PCa cells through miR-2113/MDM2 axis.	(42)
**CERS6-AS1**	PTNTs	DU145 and RWPE-1	miR-16-5p	miR-16-5p/HMGA2 axis	–	Its knockdown can prevent the proliferation and migration of DU145 cells.	(43)
**DANCR**	30 PTNTs	HPrEC, RWPE-1, PC3, DU145, LN96, and OPCT-1	miR-33b-5p	Glucose Metabolism	–	DANCR affects the proliferation, migration, and taxol resistance of PCa cells.	(44)
**DANCR**	53 PCa patients and 47 healthy persons	DU145, 22Rv1, RC-92a, PC-3M, and RWPE-1	miR-214-5p	TGF-β signaling pathway	Poor prognosis	Elevated expression of DANCR can facilitate PC progression.	(45)
**DANCR**	40 paired PCa tissues and ANTs	5 PCa cell lines and 1 epithelial cell line	miR-185-5p	FAK/PI3K/AKT/GSK3β/Snail pathway	–	DANCR exerts its oncogenic effects *via* miR-185-5p/LASP1 axis in prostate cancer.	(46)
**MALAT1**	98 paraffin-embedded clinical specimens (3 normal samples and 95 cancer tissues)	C-3, C4-2, and RWPE-1	MYBL2	MALAT1/MYBL2/mTOR Axis	–	Its knockdown inhibits the expression of p-mTOR.	(47)
**MALAT1**	52 PTNTs	RWPE-1, PC-3, and DU145	miR-140 and BIRC6	miR-140/BIRC6 axis	Poor OS	MALAT1 silencing suppresses PC progression.	(48)
**MALAT1**	–	DU145, PC3, and LNCaP	miR-423-5p	–	Decreased survival	MALAT-1 expression affects progression and survival of PCa patients.	(49)
**MALAT1**	gene expression profiles of PC patients from TCGA database	LNCaP and CWR22Rv1	miR-145	miR-145-5p-SMAD3/TGFBR2 axis		Long ncRNA MALAT1 enhances the proliferation, migration, and invasion by acting as a ceRNA for miR-145.	(50)
**MALAT1**	602 urine samples from patients with PCa and BPH	–	–	–	–	MALAT-1 and PCA3 may serve as noninvasive exosomal markers for detection of PCa.	(51)
**PCA3**							
**PCGEM1**	26 PTNTs	LNCAP, 22RV1, MDA-PCA-2B, and RWPE1	miR-129-5p	PCGEM1/miR-129-5p/CDT1 axis	–	PCGEM1 promotes the progression of PCa through sponging miR-129-5p.	(52)
**PCGEM1**	50 PTNTs	PC-3, LNPCa, Du-145, C4-2B, and RWPE1	miR-506-3p	miR-506-3p/PCGEM1/TRIAP1 axis	Distant metastasis	Facilitates the proliferation, invasion, and migration through sponging miR-506.	(52)
**NEAT1**	RNA sequencing data from TCGA and GEO databases	PC3	LDHA	–	–	NEAT1 regulates LDHA expression	(13)
**NEAT1**	130 PTNTs	–	–	–	Distant metastasis, TNM stage, and lymph nodes metastasis	It has been reported that NEAT1 plays a role in the prognosis of PCa patients.	(53)
**NEAT1**	50 PTNTs	RWPE-1, PC3, P4E6, LNCaP, and DU145	miR-766-5p	miR-766-5p/E2F3 axis	–	NEAT1 promotes progression of PCa.	(54)
**NEAT1**	plasma of 15 PCa patients and 15 HCs and 8 FFPE tissues of PCa and ANTs	–	–	–	–	NEAT1 acts as an oncogene in PCa development.	(55)
**NEAT1–1**	FFPE or fresh-frozen hormone-naïve primary prostate cancer and bone metastatic tissues (n=60)	PDXs related primary cells	CYCLINL1 and CDK19	CYCLINL1/CDK19/NEAT1-1 axis	Poor prognosis	NEAT1 induces bone metastasis of PCa *via* N6-methyladenosine.	(56)
**LINC00624**	PCa tissues	–	TEX10	LINC00624/TEX10/NF-κB axis	Poor prognosis	LINC00624 plays an oncogenic role in PCa progression.	(57)
**TP73-AS1**	–	DU-145 and PC-3 cells	TP73	TP73/TP73-AS1 axis		Knockdown of TP73-AS1 suppresses the proliferation of PCa cells by TP73 regulation.	(58)
**LINC01207**	–	PC-3, LNCaP, Du-145, C4-2B, and RWPE1	miR-1182	miR-1182/AKT3 axis	Poor prognosis	LINC01207 could directly binds with miR-1182.	(59)
**PCAT14**	499 PCa samples and 52 adjacent normal tissue samples	–	–	immune pathways	–	PCAT14 is a potential diagnosis marker in case of PCa.	(60)
**DLEU2**	Prostate tumor tissues from TCGA database	PC-3 and DU145	miR-582-5p	miR-582-5p/SGK1 axis	Poor prognosis	High expression of DLEU2 promotes the proliferation invasion, and migration of PCa cells.	(61)
**BCAR4**	90 PTNTs	PC346, LNCap, MDAPC1 2a/b, C4-2, PC3, BPH1, and DU145	miR-15 and miR-146	GLI2 signaling	–	Beclin-1 expression is regulated by BCAR4 *via* miR-146 and miR-15 in PC cells.	(62)
**EIF3J-AS1**	36 PTNTs	PC-3, LNCaP, DU-145, and RWPE-1	MAFG	–	–	EIF3J-AS1 induces progression of PCa through interaction with MAFG.	(63)
**ZEB2-AS1**	PTNTs and BPH tissues	–	–	apoptosis	–	No significant association was reported between the relative expression of this lncRNA and the tumor grade.	(64)
**HOXD-AS1**	36 and 9 cases paraffin embedded PCa and BPH tissues	LNCaP, PC-3, LNCaP-Bic, and LNCaP-AI	miR-361-5p	miR-361-5p/FOXM1 axis	High volume disease	Exosomal lncRNA HOXD-AS1 enhances distant metastasis.	(65)
**HOXA11-AS**	25 PTNTs	RWPE-1, PC-3, Du-145, and LNCaP	miR-24-3p	HOXA11-AS/miR-24-3p/JPT1 axis	–	HOXA11-AS1 functions as ceRNA for microRNA-24-3p, and regulates Jupiter microtubule associated homolog 1.	(66)
**HOXA-AS2**	68 PTNTs	RWPE, LNCaP, DU145 and PC3	miR-509-3p and PBX3	miR-509-3p/PBX3 axis	Advanced stages	Its knockdown inhibits the proliferation and migration.	(67)
**LncAY927529**	exosomes derived from PCa patient serum	BPH-1, RWPE-1, VCaP, LNCaP, DU145, and PC3	CXCL14	–	–	Exosomal lncRNA lncAY927529 induces proliferation and invasion of PCa cells.	(66)
**HCG18**	–	PC cells	miR-370-3p	miR-370-3p/DDX3X Axis	–	HCG18 promotes cell proliferation, invasion, and migration of PCa.	(68)
**LINC00115**	24 PTNTs	PC‐3, DU145, LNCap, 22RV2, and RWPE	miR-212-5p	miR-212‐5p/FZD5/Wnt/β‐catenin axis	Poor prognosis	LINC00115 acts as a ceRNA for miR-212-5p, and regulates FZD5 level.	(69)
**FOXD1-AS1**	–	RWPE-1, LNCap, PC3, and DU145	miR-3167	miR-3167/YWHAZ axis	–	FOXD1-AS1 induces malignant phenotype of PCa cells through regulating the miR-3167/YWHAZ axis.	(70)
**AC245100.4**	PCa tissues	PCa cells	–	STAT3/NR4A3 axis	–	Its silencing suppresses the tumorigenesis of PCa cells by regulating STAT3/NR4A3 axis.	(62)
**LNC992**	Gene expression microarray data from the GEO database and cancer tissues from PCa patients	PCa cells	EIF4A3		–	LNC992 enhances the growth and metastasis of PCa cells by regulating SOX4 expression.	(71)
**PCBP1-AS1**	4 BPH patients, 28 HSPC patients, and 12 CRPC patients	LNCaP and C4-2 cells	NTD domain of AR	ubiquitin–proteasome degradation process of AR	Poor prognosis	It has been reported that PCBP1-AS1 expression was significantly increased in CRPC.	(62)
**CCAT1**	10 PTNTs	RWPE-1, LnCaP, DU145, PC3, and 22RV1	miR-490-3p	miR-490-3p/FRAT1 axis	–	CCAT1 enhances the proliferation, migration, and invasion of PCa cells.	(72)
**CCAT1**	30 PTNTs	RWPE-1, PC3, and DU145	miR-24-3p and FSCN1	CCAT1/miR-24-3p/FSCN1 axis	–	CCAT1 affects the sensitivity of PCa cells to PTX by regulating miR-24-3p and FSCN1.	(73)
**LOC100996425**	110 PTNTs	C4-2, PC‐3, 22RV1, LNCap, DU‐145, and WPMV‐1	HNF4A	AMPK/mTOR signaling pathway	Lower overall survival rate	LOC100996425 serves as a promoter in PCa by modulating the AMPK/Mtor signaling pathway.	(72)
**OGFRP1**	Docetaxel-sensitive (n = 70) and docetaxel-resistant (n = 72) PCa tissues	PC3 and DU-145 and corresponding normal control PrEC prostate epithelial cells	miR-149-5p	OGFRP1/miR-149-5p/IL-6 axis	Poorer overall survival	It was reported that OGFRP1 was upregulated in docetaxel-resistant PC tissue samples in comparison to samples from docetaxel-sensitive patients.	(74)
**AATBC**	86 PTNTs	LNCaP, DU145, 22RV1, VCaP, PC3, and RWPE-1	miR-1245b-5p	miR-1245b-5p/CASK Axis	–	AATBC promotes prostate cancer progression.	(74)
**AGAP2-AS1**	–	PCa cells	miR-628-5p	AGAP2-AS1/miR-628-5p/FOXP2 axis and WNT pathway	–	AGAP2-AS1 enhances PCa cell growth by modulating WNT pathway.	(75)
**PCAT6**	CRPC tissues (n=17) and NEPC tissues (n=9)	NE-like cells (PC3, DU145, and NCI-H660), LNCaP, C4-2	miR-326	PCAT6/miR-326/Hnrnpa2b1 signaling	–	It has been reported that PCAT6 was upregulated in NE-like cells (PC3, DU145, and NCI-H660) in comparison to androgen-sensitive LNCaP cells.	(74)
**PCAT6**	20 PTNTs	–	IGF2BP2	PCAT6/IGF2BP2/IGF1R axis	Poor prognosis	The mentioned lncRNA was upregulated in tumor tissues with bone metastasis, and may act as a potential prognostic marker and therapeutic target in case of PCa patients with bone metastasis.	(76)
**CRNDE**	25 PTNTs	RWPE-1, LNCaP, PC3, DUL145, and VCaP	miR-146a-5p	–	–	CRNDE knocking down suppresses PC cells proliferation.	(71)
**LncRNA NCK1-AS1**	116 PTNTs	WPMY-1, PC-3, LNCaP, 22Rv1, and DU145	–	–	Poor prognosis	lncRNA NCK1-AS1 is upregulated in PCa. its silencing can suppress PCCs proliferation.	(76)
**AFAP1-AS1**	30 PTNTs	HprEC, PC3, and DU145	miR-195-5p	miR-195-5p/FKBP1A axis	–	AFAP1-AS1 affects the sensitivity of PCa cells to paclitaxel.	(77)
**AFAP1-AS1**	–	C4-2 cells and NE-like cells (PC3, DU145, and NCI-H660)	miR-15b	miR-15b/IGF1R Axis	–	Its expression was upregulated in castration-resistant C4-2 cells and NE-like cells, in comparison to androgen-sensitive LNCaP cells.	(74)
**LINC00467**	22 PTNTs	CaP, LNCaP, 22RV1, PC3, DU145, HrPEC, and RWPE-1	miR-494-3p	M2 macrophage polarization, STAT3 pathway and miR-494-3p/STAT3 Axis	–	Downregulation of LINC00467 prevents migration and invasion of PCa cells.	(78)
**LINC01194**	62 PTNTs	RWPE-1, PC3, DU145, and LNCap	PAX5, miR-486-5p	LINC01194/miR-486-5p/GOLPH3 axis	–	LINC01194 serves as a tumor promotor, and enhances progression of PCa by regulating LINC01194/miR-486-5p/GOLPH3 axis.	(79)
**PlncRNA-1**	34 PTNTs	DU145 and 22Rv1	–	PTEN/Akt pathway	–	PlncRNA-1 facilitates PCa cells proliferation, migration and invasion.	(80)
**MIR4435-2HG**	–	WPMY-1, VCaP, LNCaP, DU145, and PC-3	ST8SIA1	FAK/AKT/β-catenin signaling pathway	–	MIR4435-2HG affects the clone formation aptitude, proliferation, invasion, and migration of PC-3 cells.	(81)
**PTV1**	PVT1 RNA-Seq data from TCGA-PRAD database	–	–	–	Worse prognosis	PTV1 is a potential diagnosis and prognosis marker in PCa.	(74)
**PTV1**	–	DU 145, PC-3, and RWPE-1	miR-15b-5p, miR-27a-3p, miR-143-3p, miR-627-5p, and NOP2	PVT1-NOP2 axis	–	PVT1 induces metastasis in PCa.	(82)
**PVT1**	25 PTNTs	22RV1, DU145, RWPE-1, and 293T	miR-15a-5p and KIF23	PVT1/miR-15a-5p/KIF23 axis	–	PVT1 modulates KIF23 *via* miR-15a-5p.	(83)
**LINC01116**	–	RWPE-1, DU145, PC3, LNCAP, 22RV1, and VCaP	miR-744-5p	miR-744-5p/UBE2L3 axis	–	LINC01116 enhances the proliferation, migration, invasion and EMT progress of PCa cells.	(84)
**PAINT**	tissue microarray samples from normal prostate and prostate adenocarcinoma from stages I, II, III and IV	PC-3, C4-2B, 22Rv1, LNCaP-104S, and MDA-PCa-2b	Slug, Vimentin, E-cadherin	epithelial mesenchymal transition (EMT) and apoptosis	Aggressive PCa	PAINT functions as an oncogene in PCa.	(85)
**PTTG3P**	CRPC tissues and tumor tissues of patients with hormone-naive PCa	androgen-independent PC cell lines and androgen-dependent PCa cell line LNCaP	miR-146a-3p, PTTG1	–	–	PTTG3P is the ceRNA of miR-146a-3p to increase PTTG1 expression in the progression to CRPC.	(86)
**NORAD**	74 PTNTs	22Rv1, DU145, PC-3, RWPE-1, C4-2B, HS-5, and HEK293T	miR-541-3p	NORAD/miR-541-3p/PKM2 axis	–	NORAD functions as a ceRNA of miR-541-3p to enhance the expression of PKM2, leading to development of bone metastasis in PCa.	(87)
**NORAD**	45 PTNTs	RWPE-1, PC-3, LNCap, 22RV1, and DU-145	miR-30a-5p and RAB11A	miR-30a-5p/RAB11A/WNT/β-catenin pathway	–	NORAD facilitates the proliferation, invasion, EMT, and suppresses apoptosis of PCa cells.	(88)
**NORAD**	30 PTNTs	DU145, 22Rv1, LNCaP, and RWPE-1	miR-495-3p and TRIP13	miR-495-3p/TRIP13 axis	–	NORAD sponges with miR-495-3p, and increases malignant features of PCa cells.	(89)
**KCNQ1OT1**	30 PTNTs	DU145 and LNCaP	miR-211-5p	miR-211-5p/CHI3L1 Pathway	–	lncRNA KCNQ1OT1serves as a ceRNA of miR-211-5p, and upregulates CHI3L1 levels.	(90)
**KCNQ1OT1**	30 PTNTs	DU145 and PC-3	miR-15a	Ras/ERK signaling	–	KCNQ1OT1 induces immune evasion and malignant phenotypes of PC by sponging miR-15a.	(89)
**BLACAT1**	42 PTNTs	DU145, LNCap, PC-3, and RWPE-1	miR-29a-3p and DVL3	miR-29a-3p/DVL3 Axis	–	BLACAT1 facilitates the proliferation, migration and invasion of PCa cells.	(91)
**FAM83H-AS1**	8 normal prostate tissues and 20 PCa tissues	PCa cells	miR-15a	AR signaling and miR-15a/CCNE2 Axis	–	FAM83H-AS1 plays an oncogenic role in PCa, and affects cell proliferation and migration.	(92)
**RAMS11**	42 PTNTs	RWPE-2, LNCap, PC3 and DU145	CBX4	–	Poorer OS and DFS	RAMS11 enhances the growth and metastasis of PCa cells.	(86)
**AC245100.4**	–	RWPE1, DU145, PC3, and 293T	miR-145-5p and RBBP5	AC245100.4/miR-145-5p/RBBP5 axis	–	AC245100.4/miR-145-5p/RBBP5 ceRNA network promotes PCa cells development.	(90)
**Linc00662**	PTNTs	WPMY-1, PC-3, and DU145	–	–	Lymph node metastasis and distant metastasis	Linc00662 affects PCa cells proliferation, migration, invasion, and apoptosis.	(93)
**HOTAIRM1**	–	PC3 and RWPE-1	Bad, Bax, Bid, and Bcl-2	Wnt pathway	–	HOTAIRM1 suppresses the progression of PCa.	(90)
**LEF1-AS1**	AIPC samples from 45 patients	AIPC cell lines PC3, DU145, and RWPE	miR-328	Wnt/β-catenin pathway	–	LEF1-AS1 enhances the proliferation, migration, and invasion of AIPC cells through its angiogenic activity.	(94)
**PCAL7**	104 PTNTs	LNCaP and VCaP cells	HIP1	AR signaling	–	PCAL7 acts as an oncogene in PCa.	(95)
**LINC00852**	Data from TCGA database	PC-3, VCaP and androgen-stimulated LNCaP cell lines	epithelial-mesenchymal transition-related proteins	EMT	–	Its upregulation promotes PC3 cells proliferation and colony formation abilities.	(96)
**AGAP2-AS1**	50 PCa tissues and 20 BPH tissues	VCaP, 22Rv1, CRL-1740, CRL-2422, PC3M, and WPMY-1	miR-195-5p and PDLIM5	–	–	AGAP2-AS1 affects the proliferation, migration, and invasion.	(97)
**LINC01006**	–	RWPE-1, DU145, PC3, LNCAP, and VCaP	miR-34a-5p and DAAM1	LINC01006/miR-34a-5p/DAAM1 axis	–	LINC01006 serves as a ceRNA for miR-34a-5p, and up-regulate DAAM1 levels.	(92)
**MCM3AP-AS1**	64 PTNTs	PC-3, DU145, 22RV1, LNCaP, and WPMY-1	miR-543-3p	miR-543-3p/SLC39A10/PTEN axis	–	MCM3AP-AS1 induces PCa cells proliferation and invasion.	(98)
**DLX6-AS1**	20 PTNTs	WPMY1, LNCap, DU145, PC-3, and VCap	miR-497-5p and SNCG	miR-497-5p/SNCG pathway	–	DLX6-AS1 exerts oncogenic role in PCa.	(99)
**LINC00173**	124 PTNTs	RWPE-1, DU145, PC-3, and LNCap	miR-338-3p	LINC00173/MiR-338-3p/Rab25 Axis	Reduced patient survivals	LINC00173 inhibits PCa cells proliferation, migration and invasion, and enhances apoptosis.	(100)
**NNT-AS1**	–	LNCaP clone FGC, VCaP, LNCaP C4-2B, PC3, and RWPE-1	miR-496 and DDIT4	NNT-AS1/miR-496/DDIT4 regulatory axis	–	NNT-AS1 acts as the sponge of miR-496 in PCa, and upregulates DDIT4 expression.	(101)
**UCA1**	40 PTNTs	RWPE1, 22RV1, and DU145	miR-331-3p and EIF4G1	UCA1/miR-331-3p/EIF4G1 axis	–	Its knockdown increases PCa cells radiosensitivity.	(100)
**UCA1**	86 PTNTs	DU145, PC-3, LNCaP, 22Rv1, and RWPE-1	miR-143 and MYO6	UCA1/miR-143/MYO6 axis	–	UCA1 plays an oncogenic role in prostate cancer.	(102)
**IDH1-AS1**	20 PTNTs	PC3, DU145, LNCaP, 22RV1, and WPMY-1	–	IDH1-AS1-IDH1 axis	–	IDH1-AS1 is a potential target for treatment of PCa.	(103)
**CCAT2**	18 PTNTs	PCa, PC3, DU145, and RWPE-1	TCF7L2 and microRNA-217	Wnt/β-catenin signaling pathway	–	CCAT2 sponges with miR-217 to regulate TCF7L2 levels.	(98)
**AC245100.4**	42 PTNTs	RWPE-1, DU145, PC3, 22RV1, and LNCaP	HSP90	NFκB signaling pathway	–	AC245100.4 is located in cytoplasm of PCa cells.	(97)
**LINC00992**	60 PTNTs	RWPE-1, PC3, LNCaP, DU145, and C4–2	miR-3935 and GOLM1	–	–	LINC00992 promotes the proliferation and migration of PCa cells, and inhibits apoptosis.	(92)
**LINC00675**	9 primary PCa tissues and 8 CRPC tissues	LNCaP-SF and LNCaP-JP human PCa cells	GATA2	LINC00675/MDM2/GATA2/AR signaling axis	–	Expression of LINC00675 was elevated in CRPC patients.	(104)
**LINC01207**	62 PTNTs	PC-3, DU145, and RWPE-1	miR-1972 and LASP1	LINC01207/miR-1972/LASP1 axis	–	LINC01207 serves as a tumor promoter in PCa.	(105)
**MCM3AP-AS1**	30 PTNTs	PrSC cell, C4-2, PC-3, LNCaP, DU145, and 22Rv1	WNT5A and miR-876-5p	MCM3AP-AS1/miR-876-5p/WNT5A axis	Poor prognosis	MCM3AP-AS1 partakes in PCa progression.	(94)
**LINC00920**	125 prostate tumor and 10 normal tissue samples	RWPE-1, LNCaP, VCaP, DU145, and PC-3	ERG and 14-3-3ϵ protein	FOXO signaling pathway	–	LINC00920 facilitates the interaction between14-3-3ϵ protein and FOXO1.	(106)
**lncAMPC**	32 primary PCa tissues from patients undergoing radical prostatectomy and 157 urine samples from patients with positive prostate biopsy	PC-3 and RM-1 prostate cells	LIF and miR-637	lncAMPC/LIF/LIFR axis	–	lncAMPC enhances PCa cells proliferation, viability, migration, and invasion abilities.	(94)
**LINC00689**	80 PTNTs	RWPE1, DU145, LNCaP, PC-3 and C42B	miR-496 and CTNNB1	Wnt pathway	Short OS time	LINC00689 involves in progression of prostate cancer by increasing CTNNB1 levels.	(107)
**LINC00473**	–	DU145, LNCaP, PC-3, and P69	miR-195-5p and SEPT2	JAK-STAT3 signaling pathway and miR-195-5p/SEPT2 axis	–	LINC00473 partakes in PCa cell proliferation through JAK-STAT3 signaling pathway.	(108)
**FAM66C**	Prostate carcinoma dataset of the TCGA	DU145, LNCaP, PC-3, PC-3M-IE8, and WPMY-1	–	EGFR-ERK signaling, proteasome and lysosome pathways	Shorter OS	Its upregulation induces cell growth in PCa cells.	(109)
**OGFRP1**	57 PTNTs	PC-3, DU-145, C4-2, VCAP, RWPE-1, and 293T	miR-124-3p and SARM1	–	TNM stages III and IV and perineural invasion	OGFRP1 sponges with miR-124-3p, and induces PCa cells growth.	(110)
**TUG1**	39 PTNTs	RWPE-1, PC-3, and DU145	miR-496	miR-496/Wnt/β-catenin pathway	–	TUG1 sponges with miR-496, thus suppressing expression of miR-496.	(111)
**TUG1**	50 PTNTs	WPMY-1, LNCaP, 22RV1,PC-3, and DU145	miR-139-5p and SMC1A	TUG1/miR-139-5p/SMC1A axis	Lower survival rate and poor prognosis	TUG1 partakes in prostate cancer radio-sensitivity.	(92)
**TUG1**	–	RWPE1, PC-3, and DU145	Nrf2, HO-1, FTH1, and NQO1	Nrf2 signaling axis	–	TUG1 exerts oncogenic role in PCa cells.	(111)
**TUG1**	30 PTNTs	PC-3, DU145, and RWPE-1	miR-128-3p and YES1	miR-128-3p/YES1 axis	Poor prognosis	TUG1 may serves as a potential target for treatment of prostate cancer patients.	(112)
**SOX2-OT**	27 PTNTs	NPrEC. LNCaP, and DU145	HMGB3 and miR-452-5p	miR-452-5p/HMGB3 Axis and Wnt/β-Catenin Pathway	lymph metastasis, and TNM stages	SOX2-OT sponges with miR-452-5p, and modulates HMGB3 levels, and regulates the Wnt/b-catenin signaling pathway.	(105)
**LINC00665**	41 PTNTs	LNCaP, PC-3, DU-145, 22RV1, and RWPE-1	miR-1224-5p and SND1	miR-1224-5p/SND1 pathway	Poor prognosis	Its knockdown inhibits the migration and invasion of PCa cells.	(113)
**ZEB1-AS1**	30 PTNTs	RWPE-1, DU145, and LNCaP	miR-342-3p and CUL4B	PI3K/AKT/mTOR signal pathway and miR-342-3p/CUL4B axis	–	ZEB1-AS1 silencing represses PCa cells proliferation, migration, and invasion.	(110)
**UNC5B-AS1**	50 PTNTs	PC-3, DU-145, 22RV1, Lncap and WPMY-1	caspase-9	–	Distant metastasis and advanced pathological stage	UNC5B-AS1 regulates the expression of Caspase-9 in PCa tissues and cell lines.	(114)
**CRNDE**	64 PTNTs	PC3 and 22RV1	miR-101	miR-101/Rap1A axis	Poor outcomes	Increased CRNDE levels induces the proliferation, migration, and invasion of Pca cells.	(110)
**ZFAS1**	30 PTNTs	RWPE-1, PC3, DU145, 22RV1, and LNCAP	miR-135a-5p	–	–	ZFAS1 silencing suppresses PCa cell proliferation, invasion, and metastasis through modulating miR-135a-5p.	(115)
**PRRT3-AS1**	GSE55945 and GSE46602 datasets	DU145, LNCaP, PC3, IA8, IF11, and RWPE-1	PPARγ	mTOR signalling pathway	–	Its silencing suppresses the mTOR signaling pathway.	(116)
**LINC00673**	48 PTNTs	PC3, LNCap, DU145, paclitaxel-resistant cell line (DU145/pr), and RWPE-1	KLF4	–	TNM stage and LNM	LINC00673 modulates KLF4.	(117)
**VPS9D1-AS1**	PRAD tissues from TCGA database	RWPE-1, DU145, VCaP, PC-3, and LNCaP	miR-4739, ZEB1 and MEF2D	miR-4739/MEF2D axis	–	VPS9D1-AS1 enhances the proliferation, migration, and invasion.	(116)
**NCK1-AS1**	Blood samples from 60 patients with PCa, 58 patients with BPH, and 60 healthy males	DU145, 22Rv1, and RWPE-1	TGF-β1	TGF-β pathway	–	Expression of NCK1-AS1 was elevated in plasma of PC patients in comparison to patients with BPH and healthy controls.	(118)
**VIM-AS1**	88 PCa and 31 normal prostate tissue samples	RWPE-1, LNCaP, DU145, 22RV1, and PC3	vimentin	EMT	Large tumor size, metastasis and advanced TNM stage	Expression of VIM-AS1 affects the migration and invasion of PCa cells.	(119)
**MALAT1**	10 pairs of PCa tissues and ANTs	DU145 and 22RV1	METTL3	PI3K/AKT signaling pathway	Tumor recurrence	Elevated level of MALAT1 results in tumor recurrence in PCa patients.	(120)
**MAFG-AS1**	495 PCa tissues and 50 ANTs	PC-3 and DU145	ribosome-related genes	ribosome and DNA replication pathways	Poor prognosis	MAFG-AS1 silencing suppresses the proliferation, migration, and invasion of PCa CELLS.	(121)
**lncRNA AC008972.1**	PCa tissues	PC3 and LNCaP	miR-143-3p	lncRNA AC008972.1/miR-143-3p/TAOK2 axis	Low OS	AC008972.1 plays an oncogenic role in the progression of PCa and may serve as a possible therapeutic target in case of PCa.	(122)

BPH, benign prostate hyperplasia; PCa, prostate cancer; PTNTs, paired tumor-non-tumor tissues; HSPC, hormone-sensitive prostate cancer; CRPC, castration-resistant prostate cancer.

In addition, MALAT1 has been found to regulate glucose metabolism through modulation of MYBL2/mTOR axis (47). Moreover, *in vitro* and *in vivo* studies have shown the importance of MALAT1/miR-140/BIRC6 axis in the progression of prostate cancer (48). In fact, MALAT1 acts as a molecular sponge for miR-140 to enhance expression of the anti-apoptotic protein BIRC6 (48). In turn, expression and activity of MALAT1 have been shown to be regulated by miR-423-5p, a miRNA that impedes activity of MALAT1 in enhancement of proliferation, migration, and invasiveness of prostate cancer cells (49). Most importantly, up-regulation of miR-423-5p could enhance survival and decrease metastasis formation in a xenograft model of prostate cancer (49). In addition, MALAT1 has a possible diagnostic value in prostate cancer. Expression levels of PCA3 and MALAT1 in urinary exosomes have been shown to be superior to the currently used clinical parameters in detection of prostate cancer, particularly high-grade ones (51).

NEAT1 has also been shown to regulate aerobic glycolysis to affect tumor immunosurveillance by T cells in this type of cancer (13). It can also promote progression of prostate cancer through modulation of miR-766-5p/E2F3 axis (54).

CTBP1-AS is reported as the antisense-RNA transcript positively regulated by androgen and promotes castration-resistant prostate cancer tumor growth (123). This lncRNA is localized in the nucleus and its levels are mostly increased in prostate cancer. It enhances both hormone-dependent and castration-resistant tumor growth. From a mechanistical point of view, CTBP1-AS suppresses the expression of CTBP1 through recruitment of PSF and histone deacetylases. It also exerts androgen-dependent function through inhibition of tumor-suppressor genes and enhancement of cell cycle progression (123).

Epigenetic repression of AR corepressor is an important mechanism for AR activation. ARLNC1 is also regulated by androgen and upregulates AR mRNA stability by binding to the 3’-UTR. In line with this, ARLNC1 silencing leads to inhibition of AR expression and suppression of AR signaling as well as of growth of prostate cancer. In fact, ARLNC1 has a role in the preservation of a positive feedback loop that induces AR signaling in the course of prostate cancer progression (124). In addition to these lncRNAs, several CRPC-specific AR-regulated lncRNAs are important for overexpression of AR and its variant. These AR-regulated lncRNAs are over-expressed in CRPC tissues. An experiment in these cells has shown that knock-down of PRKAG2-AS1 and HOXC-AS1 leads to suppression of CRPC tumor growth in addition to inhibition of expression of AR and AR variant. Mechanistically, PRKAG2-AS1 modulates the subcellular localization of the splicing factor, U2AF2. This splicing factor is involved in the AR splicing system (125).

SChLAP1 is another up-regulated lncRNA in prostate cancer whose up-regulation is associated with poor patient outcomes, such as metastases and prostate cancer specific mortality. It has a critical role in invasiveness and metastasis. Functionally, SChLAP1 influences the localization and regulatory function of the SWI/SNF complex (126).

PCAT-1 is another up-regulated lncRNA in prostate cancer which enhances cell proliferation through cMyc. Mechanistically, PCAT-1–associated proliferation depends on stabilization of cMyc protein. Moreover, cMyc has an essential role in a number of PCAT-1–induced expression alterations (127).

HOTAIR as regarded as an AR-repressed lncRNA is upregulated after androgen deprivation therapy and in CRPC. Mechanistically, HOTAIR binds to the AR protein to inhibit its interactions with the E3 ubiquitin ligase MDM2, thus suppressing AR ubiquitination and its degradation. Therefore, HOTAIR induces androgen-independent AR activation and drives the AR-mediated transcriptional program in the absence of androgen (128). Another study has shown that NEAT1 induces oncogenic growth in prostate tissue through changing the epigenetic marks in the target genes promoters to induce their transcription (129). Moreover, PCGEM1 and PRNCR1 bind to AR and enhance selective looping of AR-bound enhancers to target gene promoters (130). Similarly, SOCS2-AS1 interacts with AR for co-factor interaction (131).

**Figure 1:**
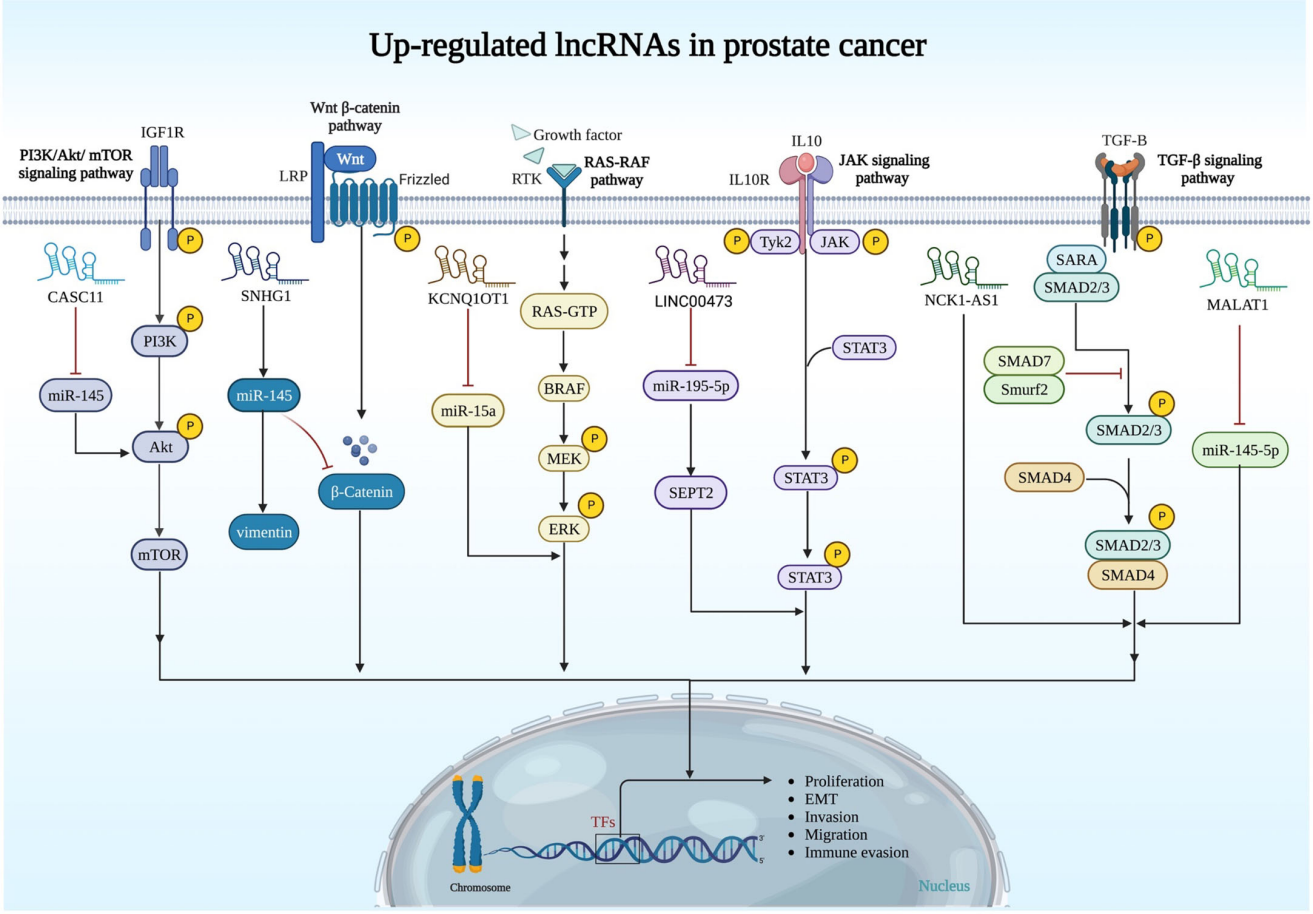
Upregulation of oncogenic lncRNAs and their relation with signaling pathways in prostate cancer. PI3K/AKT/mTOR, Wnt/β-catenin, RAS/RAF, JAK and TGF-β pathways are regulated by oncogenic lncRNAs in prostate cancer.

The importance of other up-regulated lncRNAs in prostate cancer is summarized in **Figure 1** and **Table 1**.

## Down-regulated lncRNAs in prostate cancer

**Figure 2:**
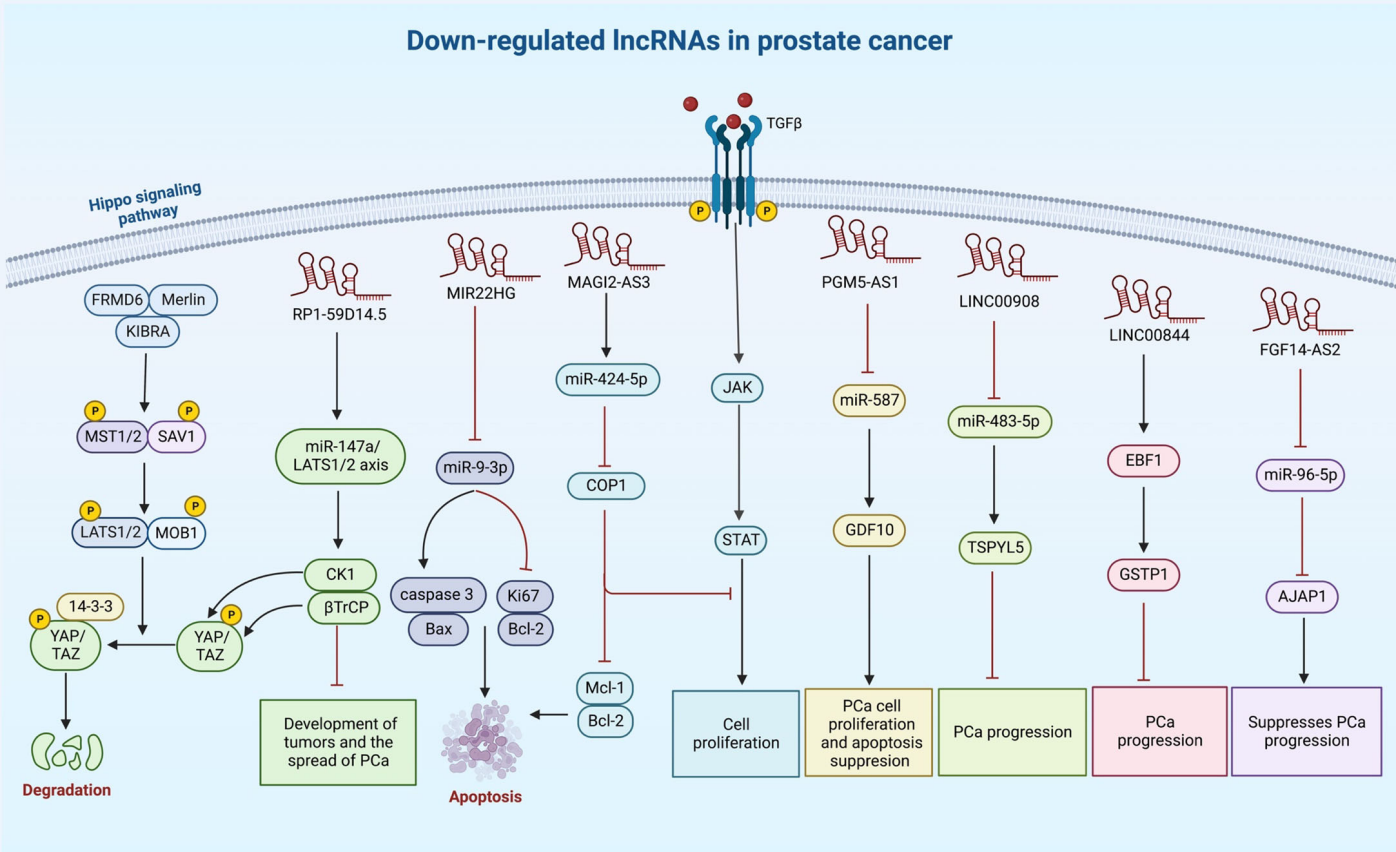
A synopsis of the known roles of lncRNA tumor suppressors in prostate cancer. Several lncRNAs can reduce cell proliferation and invasiveness of prostate cancer cells, particularly through sponging oncogenic miRNAs.

A number of other lncRNAs have been found to act as tumor suppressors in prostate cancer (**Table 2**). For instance, LINC00893 can inhibit progression of this type of cancer *via* modulation of miR-3173-5p/SOCS3/JAK2/STAT3 axis (132). Similarly, the sponging effect of LINC01679 on miR-3150a-3p has a role in inhibition of progression of prostate cancer through affecting expression of SLC17A9 (133). MIR22HG is another tumor suppressor lncRNA that acts as a molecular sponge for miR-9-3p (134). The tumor suppressor role of RP1-59D14.5 in prostate cancer is mediated through activation of the Hippo signaling and enhancement of autophagy (135). Moreover, MAGI2-AS3 has been shown to inactivate STAT3 signaling and suppress proliferation of prostate cancer cells through acting as a miR-424-5p sponge (136). NXTAR is another tumor suppressor lncRNA that modulates expression of androgen receptor (AR) and resistance to enzalutamide (137). Totally, the number of identified tumor suppressor lncRNAs in prostate cancer is far below that of oncogenic lncRNAs (**Figure 2**). **Table 2** summarizes the information about tumor suppressor lncRNAs in prostate cancer.

**Table 2:** Summary of function of down-regulated lncRNAs in prostate cancer (Official HUGO Gene Nomenclature symbols are used).

lncRNA	Samples	Cell line	Targets/Regulators	Signaling Pathways	Association with patients’ outcome	Function	Ref
**LINC00893**	66 PTNTs	PC-3, DU145, VCaP, LNCaP, and RWPE-1	miR-3173-5p	miR-3173-5p/SOCS3/JAK2/STAT3 axis	Poorer overall survival rate	LINC00893 is a tumor-suppressor in PCa.	(132)
**LINC01679**	55 PTNTs	RWPE-2, DU145, PC-3, LNCaP, C4-2B, and 22RV1	miR-3150a-3p	miR-3150a-3p/SLC17A9 axis	Poor survival	LINC01679 serves as a molecular sponge for miR-3150a-3p in prostate cancer.	(133)
**MIR22HG**	–	RWPE-2, 22Rv1, DU145, LNCaP, and PC3	miR-9-3p	MIR22HG/miR-9-3p axis	–	MIR22HG reduces cell proliferation and enhances apoptosis in DU145 cells.	(134)
**RP1-59D14.5**	–	LNCaP, PC3, DU145, and RWPE-1	miR-147a/LATS1/2 axis	Hippo signaling pathway	–	RP1-59D14.5 acts as a ceRNA for miR-147a, and regulates large tumor suppressor kinase 1/2.	(135)
**MAGI2-AS3**	109 PTNTs	WPMY-1, PC-3 and DU145	miR-424-5p and COP1	STAT signaling	–	Elevated expression of MAGI2-AS3 suppresses PCa cell proliferation.	(136)
**NXTAR**	PTNTs	RWPE-1, 22Rv1, LNCaP, VCaP, PC3, LAPC4, and C4-2B	–	ACK1/AR signaling	–	NXTAR expression was lower in various AR-positive PCa cell lines in comparison to normal prostate cells.	(137)
**FGF14-AS2**	Gene expression profiles of PC patients from TCGA database	RWPE-1, DU145, PC‐3, PC‐3 M, and LNCaP	miR-96-5p	iR-96-5p/AJAP1 axis	–	lncRNA FGF14-AS2 affects proliferation and metastasis of PCa cells by regulating iR-96-5p/AJAP1 axis.	(138)
**ADAMTS9-AS1**	68 PTNTs	PC3, DU145 and Normal human prostate epithelial cells	miR-142-5p	miR-142-5p/CCND1 axis	TNM stage and perineural invasion	ADAMTS9-AS1 suppresses the progression of PCa by affecting the miR-142-5p/CCND1 axis.	(139)
**MBNL1-AS1**	Tissues of prostate adenocarcinoma (PARD) and normal tissues	LAPC4, LNCaP, DU145, C4-2B, and RWPE-1	miR-181a-5p	PTEN/PI3K/AKT/mTOR pathway	–	MBNL1-AS1 regulates PTEN by sequestering miR-181a-5p.	(140)
**LINC00641**	23 PTNTs	PC-3, C42B, LNCaP, and RWPE-1	VGLL4 and miR-365a-3p	miR-365a-3p/VGLL4 axis	Lower survival rate	LINC00641 is a tumor suppressor lncRNA in PCa, and modulates miR-365a-3p/VGLL4 axis.	(141)
**PGM5-AS1**	PCa-related microarray datasets (GSE3325 and GSE30994)	PC-3, LNCap, 22RV1, DU145, and RWPE-1	miR-587, GDF10	PGM5-AS1/miR-587/GDF10 axis	–	PGM5-AS1 acts as a ceRNA for miR-587, and upregulates GDF10 levels.	(142)
**GAS5**	51 PTNTs	DU145, LNCaP, and WPMY-1	miR-320a and RAB21	miR-320a/RAB21 axis	–	Its upregulation inhibits viability and migration of PCa cells.	(143)
**GAS5**	–	–	–	GAS5/miR-18a-5p/serine/threonine kinase 4	–	GAS5 functions as a tumor suppressor, and inhibits the metastasis and proliferation of paclitaxel-resistant PCa cells	(121)
**LINC00261**	83 PTNTs	LNCap, PC-3, DU145, 22Rv1, ARCaP, and RWPE-1	DKK3 and GATA6	LINC00261/GATA6/DKK3 axis	–	LINC00261 modulates DKK3.	(144)
**EMX2OS**	25 PTNTs	LNCaP, DU145, PC3, RWPE-1 and HEK293A	FUS and TCF12	cGMP-PKG pathway	–	EMX2OS suppresses tumor growth *in vivo*.	(145)
**LINC00844**	62 PTNTs	22Rv1, VCaP, LNCaP, Du145, PC-3, and RWPE‐1	GSTP1 and EBF1	LINC00844/EBF1/GSTP1 axis	–	LINC00844 may serve as a potential target for PCa treatment.	(146)
**Erbb4-IR**	60 PTNTs	22Rv1 and DU145	miR-21	–	Poor survival	Erbb4-IR mediates the proliferation and apoptosis of PCa cells through miR-21.	(147)
**MIR22HG**	9 normal and 13 prostate tumor sample	LNCaP, WPMY-1, PC-3 and C4-2B	–	TNF, Cytokine-cytokine receptor interaction, MAPK, NF-κB, Jak-STAT, p53, NOD-like receptor signaling, Toll-like receptor, Cytosolic DNA-sensing, and PI3K-Akt	T stage	MIR22HG may acts as a potential biomarker in case of prostate cancer diagnosis.	(148)
**FER1L4**	78 PTNTs	PC-3, LNCaP, DU145, and RWPE-1	FBXW7 and miR-92a-3p	ER1L4/miR-92a-3p/FBXW7 axis and key signaling pathway	–	FER1L4 inhibits cell proliferation and promotes cell apoptosis by increasing expression of FBXW7 in PCa cells.	(145)
**BLACAT1**	25 PTNTs	PC3, DU145, and RWPE-1	DNMT1, HDAC1, EZH2, MDM2 and miR-361	–	–	Its silencing reduces the growth of PCa cells, and induces cell death.	(102)
**LINC00908**	55 PTNTs	VCaP, LNCaP, DU-145, PC-3, and RWPE-1	miR-483-5p and TSPYL5	LINC00908/miR-483-5p/TSPYL5 axis	–	LINC00908 sponges with miR-483-5p and suppresses PCa progression.	(149)
**DGCR5**	64 PTNTs	22Rv1 and DU145	TGF-β1	–	Poor survival	High expression of DGCR5 reduces PCa cells stemness.	(150)
**MAGI2-AS3**	PCa serum samples	LNCaP and PC3 cells	miR-142-3p	–	–	High level of MAGI2-AS3 inhibits proliferation, migration, and invasion of PCa cells.	(151)

PCa, prostate cancer; PTNTs, paired tumor-non-tumor tissues.

## Contribution of lncRNAs variants in prostate cancer

Contribution of single nucleotide polymorphisms (SNPs) within *GAS5*, *POLR2E*, *MEG3*, *MALAT1* and *HOTAIR* in the risk of prostate cancer has been assessed in different ethnic groups (**Table 3**). Three SNPs within *GAS5* have been the subject of these investigations. First, rs145204276 (delCAAGG) is located within the promoter region of *GAS5*. Compared with subjects carrying ins/ins genotype, cases with ins/del or del/del genotype of this polymorphism have shown decreased risk of pathological lymph node metastasis (152). The rs17359906 in *GAS5* is another SNP whose A allele has been shown to be a risk allele for prostate cancer. Similarly, A allele of rs1951625 SNP within *GAS5* has been associated with higher risk of this cancer. Both rs17359906 G > A and rs1951625 G > A have been associated with high plasma level of PSA. Most importantly, the recurrence-free survival of patients with prostate cancer has been lowest in patients having AA genotype of rs17359906 and highest in those having GG genotype. Similar findings have been reported for the rs1951625 (153).

**Table 3:** Contribution of lncRNAs SNPs in prostate cancer.

Gene	Polymorphism	Samples	Population	Association	Ref
***GAS5***	rs145204276	Blood samples from 579 PCa patients and 579 healthy controls	Taiwan	Compared with subjects carrying ins/ins genotype, cases with ins/del or del/del genotype of this polymorphism demonstrate decreased risk of pathological lymph node metastasis.	(152)
***GAS5***	rs17359906 G > A	Blood samples from 218 PCa patients and 220 healthy controls	Chinese Han	The mentioned SNP is correlated with increased plasma PSA levels.	(153)
	rs1951625 G > A			Subjects who carry the A allele of this polymorphism show a significantly higher risk of PCa compared to those who carry the G allele.	
***POLR2E***	rs3787016	5 eligible case-control studies including 5472 cases and 6145 controls	–	Genotypes carrying the T allele of the mentioned polymorphism show an increased risk for PCa.	(154)
***MEG3***	rs11627993 C>T	Blood samples from 65 prostate cancer patients and 200 healthy subjects	Chinese Han	No statistically significant results.	(155)
	rs7158663 A>G				
***MALAT1***	rs619586	Blood samples from 579 patients with prostate cancer	Taiwan	Cases with G allele of this polymorphism have an elevated risk of being in an advanced Gleason grade group.	(156)
	rs3200401			No statistically significant results.	
	rs1194338			Subjects who carry at least one polymorphic A allele of the mentioned SNP are positively associated with node-positive PCa.	
***HOTAIR***	rs12826786	Peripheral blood samples of 128 PCa patients, 143 BPH patients and 250 normal males	Iranian	Mentioned polymorphism is associated with PCa risk in co-dominant and recessive models.	(157)
	rs1899663			T allele of this SNP is associated with BPH risk.	
	rs4759314			No statistically significant results.	

A systematic review and meta-analysis of 5 studies on the role of rs3787016 within *POLR2E* has revealed increased susceptibility to prostate cancer for carriers of T allele in all genotype models (154). The results of other studies on contribution of lncRNAs SNPs in prostate cancer are shown in **Table 3**.

## Importance of lncRNAs as prognostic factors in prostate cancer

Several studies have indicated the importance of dysregulation of lncRNAs in the prediction of survival times of patients with prostate cancer (**Table 4**). Overall, up-regulation of oncogenic lncRNAs is predictive of lower survival time of patients in terms of overall survival or progression-free survival. For tumor suppressor lncRNAs, an opposite effect has been seen.

**Table 4:** Importance of lncRNAs as prognostic factors in prostate cancer (PTNTs, paired tumor-non-tumor tissues; PCa, prostate cancer; OS, overall survival; PFS, progression-free survival).

lncRNA	Sample number	Kaplan-Meier analysis	Univariate cox regression	Multivariate cox regression	Ref
**UBE2R2-AS1**	74 PTNTs	Its high expression is associated with poorer survival rate.	–	Gleason score and expression of UBE2R2-AS1 are independent prognostic factors for OS of PC patients.	(13)
**SNHG17**	52 PTNTs	Its high expression is associated with poor BCR-free survival.	Over expression of SNHG17 is associated with poor OS in PC patients.	Its expression is an independent prognostic factor for OS in patients with PC.	(14)
**LINC00893**	66 PTNTs	Its low expression is correlated with poorer OS.	–	–	(132)
**LINC01679**	55 PTNTs	Its low expression is correlated with reduction in DFS.	–	–	(133)
**SNHG3**	30 PTNTs	Its high expression is associated with shorter OS time.	–	–	(32)
**lncHUPC1**	70 PTNTs	High lncHUPC1 expression is correlated with poor PFS.	–	–	(41)
**MNX1-AS1**	40 PTNTs	Its high expression is correlated with worse OS rates.	–	–	(42)
**NEAT1**	50 PTNTs	Its high expression is associated with lower survival rate.	–	–	(54)
**SNHG3**	50 PTNTs	Its upregulation is associated with shorter OS and BMFS.		Its high expression is an independent risk factor for death and progression in patients with PCa.	(32)
**DLEU2**	Prostate tumor tissues from TCGA database	Its high expression is correlated with lower survival rate.	Its upregulation is associated with a poor progression-free interval.	Its upregulation is independently associated with a poor progression-free interval.	(61)
**HOXD-AS1**	36 PCa and 9 BPH cases	Its high expression is associated with shorter PSA.	Serum exosomal HOXD-AS1 in conjunction with tumor stage is a prognostic factor for PRFS.	Serum exosomal HOXD-AS1 is an independent prognostic factor for PFS	(65)
**SNHG10**	gene expression profiles of PCa patients from TCGA database	Its high expression is associated with poor PFS of PC patients.	Elevated expression of SNHG10, T stage, N stage, Gleason score, primary therapy outcome, residual tumor, and PSA were associated with PFS in patients with PCa.	SNHG10 is an independent prognostic factor for PFS in PC patients	(26)
**PCBP1-AS1**	4 BPH patients, 28 HSPC patients, and 12 CRPC patients	Its high expression indicates a poor prognosis for PCa patients.	–	–	(62)
**LOC100996425**	110 PTNTs	Its elevated expression is associated with a lower OS rate of PCa patients.	–	–	(72)
**OGFRP1**	70 docetaxel-sensitive and 72 docetaxel-resistant PCa tissues	Its higher expression in docetaxel-resistant patients is associated with poorer OS relative to the docetaxel-sensitive patients.	–	–	(74)
**DANCR**	53 PTNTs	Its high expression is associated with lower OS in PCa patients.	Its expression might be prognostic indicators of PC patients.	DANCR is an independent prognostic indicator for PCa.	(45)
**SNHG17**	53 PTNTs	Its high expression is associated with poor OS time.	–	–	(16)
**PVT1**	RNA-Seq data from TCGA-PRAD database	Its high expression is associated with poor vital survival rates.	Its expression is associated with OS and relapse-free survival.	Its high expression is an independent prognostic factor for poor OS and poor relapse-free survival in PCa.	(74)
**NORAD**	74 PTNTs	Its high expression is positively associated with OS of patients with PCa.	–	–	(87)
**ADAMTS9-AS1**	68 PTNTs	Its low expression is associated with TNM stage and perineural invasion.	–	–	(139)
**RAMS11**	42 PTNTs	Its upregulation is correlated with poorer OS and DFS.	–	–	(86)
**SNHG9**	495 PCa tissues and 52 adjacent prostate tissues	Its high expression is associated with poor prognosis.	Its expression level is associated with poorer PFS.	Its expression is independently associated with PFS in PCa patients.	(27)
**LINC00641**	23 PTNTs	Its low expression is associated with lower survival rate.	–	–	(141)

## Discussion

Several lncRNAs have been shown to contribute to the pathogenesis of prostate cancer *via* modulation of AR signaling, ubiquitin–proteasome degradation process of AR or other important signaling pathways. Some of them such as PCA3 are highly specific for this kind of cancer, representing an appropriate biomarker for prostate cancer (151). Others might be over-/under-expressed in a variatey of cancers, being therapeutic targets for a wide range of human malignnacies. The observed differences in expression of some lncRNAs between castration-resistant prostate cancer and androgen deprivation therapy-responsive cases imply the importance of these transcripts in defining response of patients to this therapeutic modality and represent these transcripts as targets for management of resistance to this therapy.

Although numerous prostate cancer-specific or prostate cancer-associated lncRNAs have been recognized, few lncRNAs have been verified in independent patient cohorts or approved for using in clinical settings. The most important milestone in the field of lncRNA research is probably approval of urinary PCA3 as a biomarker for detection of prostate cancer by the United States Food and Drug Administration (158). This lncRNA is a promising factor for urine test for prostate cancer and has a superior performance compared with PSA in urinary detection of this disorder. Further reseraches are needed to find other appropriate lncRNA biomarkers for this kind of cancer.

LncRNA profiles can also been used to identify prostae cancer patients that benefit from radiotherapy. For instance, UCA1 has beens shwon to mediate radiosensitivity in prostate cancer cell lines and therefore might be a marker to predict response to radiotherapy in these patients. This lncRNA affects radiosensitivity through influencing cell cycle progression (159).

The importance of lncRNAs in the mediation of cell proliferation, invasiveness and metastasis has potentiated them as therapeutic targets for prostate cancer. The results of animal studies have been promising particularly for some AR-regulated lncRNAs. However, clinical studies are missing in this field.

Notably, LncRNAs are also involved in drug resistance in prostate cancer cells, thus they are proper candidates for therapeutic targeting (160). For instance, HORAS5 up-regulation can trigger taxane resistance in CRPC cells through upregulation of BCL2A1. HORAS5 silencing can reduce resistance of prostate cancer cells to cabazitaxel and enhance the efficacy of chemotherapy (161).

PI3K/AKT/mTOR, Wnt/β-catenin, TGF-β, p53, FAK/PI3K/AKT/GSK3β/Snail, STAT3, FAK/AKT/β catenin, Ras/ERK, NF-κB and FOXO signaling pathways are among signaling pathways that are modulated by lncRNAs in the context of prostate cancer. Moreover, several lncRNAs have been shown to act as molecular sponges for miRNAs to regulated expression of miRNA targets. miR-145/IGF1R, miR-23a/OTUB1, miR-339-5p/STAT5A/SNORA71B, miR-144/CD51, miR-5590-3p/YY1, miR-195/CCNE1, miR-184/IGF, miR-152-3p/SLC7A11, miR-214-3p/TGF-β, miR‐577/SMURF1, miR-377-3p/AKT2, miR-133b/SDCCAG3, miR-2113/MDM2, miR-16-5p/HMGA2, miR-140/BIRC6 axis, miR-145-5p-SMAD3/TGFBR2, miR-129-5p/CDT1 axis, miR-766-5p/E2F3, miR-1182/AKT3, miR-582-5p/SGK1, miR-361-5p/FOXM1, miR-24-3p/JPT1, miR-509-3p/PBX3, miR-370-3p/DDX3X, miR-212‐5p/FZD5, miR-3167/YWHAZ, miR-490-3p/FRAT1, miR-24-3p/FSCN1, miR-149-5p/IL-6, miR-1245b-5p/CASK, miR-628-5p/FOXP2, miR-326/Hnrnpa2b1, miR-195-5p/FKBP1A, miR-15b/IGF1R, miR-494-3p/STAT3, miR-486-5p/GOLPH3, miR-15a-5p/KIF23 and miR-101/Rap1A are among putative miRNA/mRNA axes that are modulated by oncogenic lncRNAs in the context of prostate cancer.

Although expression profile of lncRNAs have been comprhensively assessed in tumoral tissues of patients with prostate cancer, less effort has been made for analysis of their expression in urine or serum samples. Based on the availability of these sources for non-invasive diagnostic procedures, future studies should focus on these biofluids to facilitate early detection of prostate cancer *via* non-invasive methods.

Taken together, lncRNAs have been found to contribute to the pathogenesis of prostate cancer through various mechanisms. These transcripts can be used as targets for therapeutic interventions in this kind of cancer.

## Author contributions

MT and AB designed and supervised the study. SG-F wrote the draft and revised it. EB, BH, and AK collected the data and designed the figures and tables. All authors contributed to the article and approved the submitted version.
